# Supplementary material for: Molecular insights into the gating mechanisms of voltage-gated calcium channel CaV2.3
Source: Nat Commun. 2023 Jan 31;14:516. doi: 10.1038/s41467-023-36260-2 (PMC9889812; doi:10.1038/s41467-023-36260-2)
Supplement: Supplementary file 1 — Supplementary Information [file 41467_2023_36260_MOESM1_ESM.pdf]

Supplementary information for

Molecular insights into the gating mechanisms of voltage-gated calcium channel $Ca_V2.3$

Yiwei Gao, Shuai Xu, Xiaoli Cui, Hao Xu, Yunlong Qiu, Yiqing Wei, Yanli Dong, Boling Zhu, Chao Peng, Shiqi Liu, Xuejun Cai Zhang, Jianyuan Sun, Zhuo Huang, and Yan Zhao

This document contains Supplementary Figure 1–8 and Supplementary Table 1 and 2.

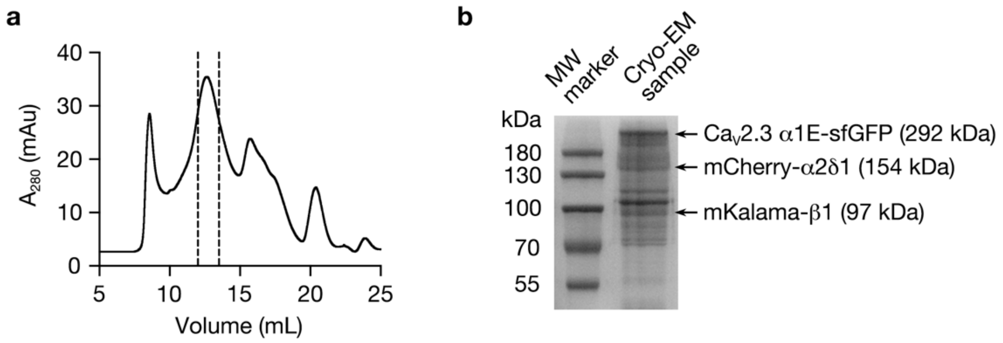

Supplementary Figure 1. Protein purification of the $Ca_V2.3$-α2δ1-β1 complex

a. Size-exclusion chromatogram of the purified protein sample of $Ca_V2.3$ complex. Peak fractions marked within the dashed lines were pooled and concentrated for the cryo-EM studies. **b**. Coomassie blue-stained SDS-PAGE gel of the purified $Ca_V2.3$ complex. Bands representing the $Ca_V2.3$ α1E, α2δ1, and β1 subunits were labeled. The experiments were repeated independently for more than 3 times with identical results.

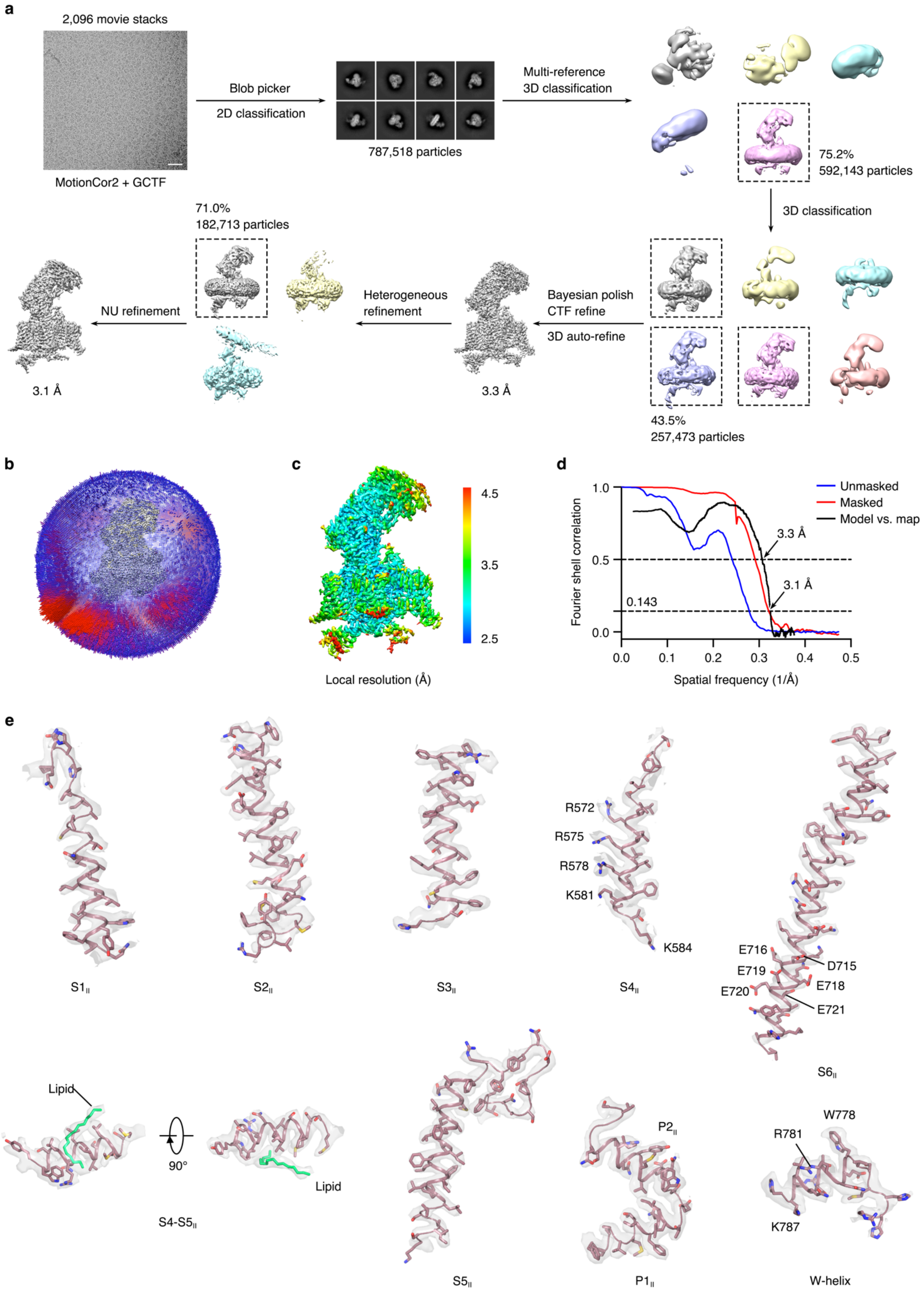
a
2,096 movie stacks
MotionCor2 + GCTF
Blob picker
2D classification
787,518 particles
Multi-reference
3D classification
75.2%
592,143 particles
3D classification
Bayesian polish
CTF refine
3D auto-refine
43.5%
257,473 particles
3.3 Å
Heterogeneous
refinement
71.0%
182,713 particles
NU refinement
3.1 Å
b
c
4.5
3.5
2.5
Local resolution (Å)
d
Fourier shell correlation
Spatial frequency (1/Å)
Unmasked
Masked
Model vs. map
3.3 Å
3.1 Å
0.143
e
$S1_{II}$
$S2_{II}$
$S3_{II}$
R572
R575
R578
K581
K584
$S4_{II}$
E716
D715
E719
E718
E720
E721
$S6_{II}$
Lipid
90°
Lipid
$S4\text{-}S5_{II}$
$S5_{II}$
$P2_{II}$
$P1_{II}$
W778
R781
K787
W-helix

Supplementary Figure 2. Cryo-EM data processing of the $Ca_V2.3$-α2δ1-β1 complex

a. Flow chart of cryo-EM data processing. A total of 787,518 particles were picked form 2,096 micrographs. A representative motion-corrected micrograph of the dataset was shown here (bar = 400 Å). A round of multi-reference 3D classification, and a further round of 3D classification against a single starting reference were performed to clean particles, followed by Bayesian polish and contrast transfer function (CTF) refinement to improve the map quality. Heterologous refinement and non-uniform (NU) refinement generated the final map, which was reported at 3.1 Å according to the golden-standard *Fourier* shell correlation (GSFSC) criterion. **b**. Angular distribution of the particles contributing to the final 3D reconstruction. The height of each spike indicated the number of particles in each designated orientation. **c**. Sharpened map of the $Ca_V2.3$ complex, colored according to the estimated value of local resolution. **d**. *Fourier* shell correlations (FSC) curves of the $Ca_V2.3$ complex and its atomic model. The unmasked (blue) and masked (red) FSC of the $Ca_V2.3$ EM map was calculated between two independently refined half-maps before and after post-processing. The model-vs-map FSC (black) was calculated between the full map and the atomic model. **e**. Representative cryo-EM density map (transparent grey surface) of the Domain II. Residues on the $S4_{II}$, $S6_{II}^{NCD}$ and W-helix were labeled. The lipid molecule residing near the S4-$S5_{II}$ was colored green and also indicated.

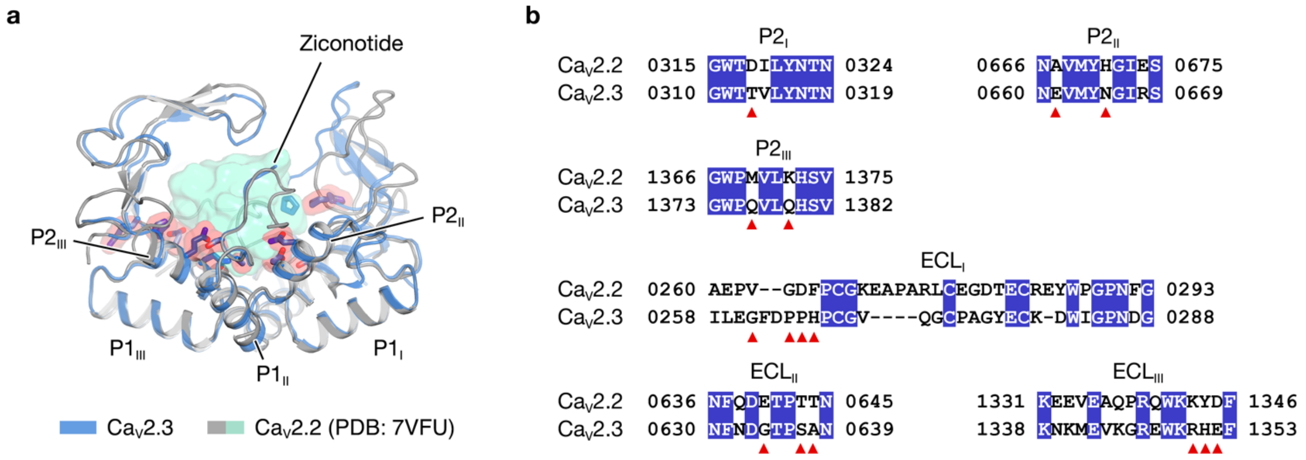

Supplementary Figure 3. Structural analysis of the ziconotide resistance of $Ca_V2.3$

a. Putative structural mismatches between the $Ca_V2.3$ and ziconotide. Extracellular helices (ECLs) and the P-loops (P1 and P2) of the $Ca_V2.3$ (blue) are superimposed on the corresponding structures of the ziconotide-bound $Ca_V2.2$. The ziconotide is shown as transparent green surface. Non-conserved residues that may contribute to the structural mismatch between $Ca_V2.3$ and ziconotide are shown as sticks, overlaid by red surfaces, and indicated in (**b**). **b**. Sequences alignments between $Ca_V2.3$ and $Ca_V2.2$ at the P-loops and ECLs. Conserved residues are highlighted in blue. Non-conserved residues that form close contact with ziconotide in $Ca_V2.2$ are indicated using red triangles.

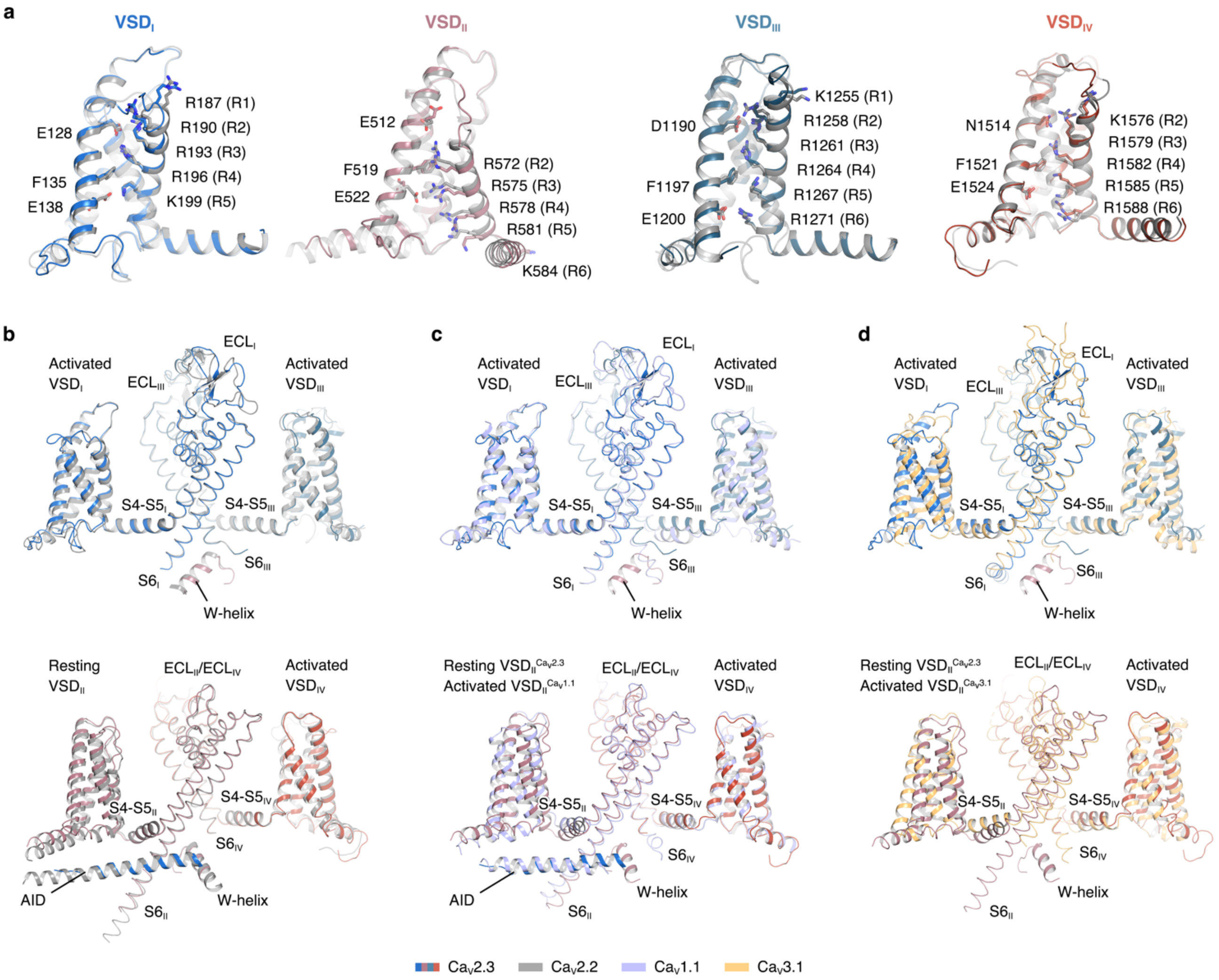

Supplementary Figure 4. VSDs among the Ca$_V$ structures

Superimpositions of the voltage-sensing domains (VSDs) or pore domains between Ca$_V$2.3 and Ca$_V$2.2 (**a**–**b**), Ca$_V$1.1 (**c**) or Ca$_V$3.1 (**d**). The VSDs were shown as cartoon, and pore domain as loops. Four domains of Ca$_V$2.3 was colored blue, pink, deep cyan, and red, respectively. The Ca$_V$1.1, Ca$_V$2.2, and Ca$_V$3.1 was colored light purple, gray, and yellow, respectively.

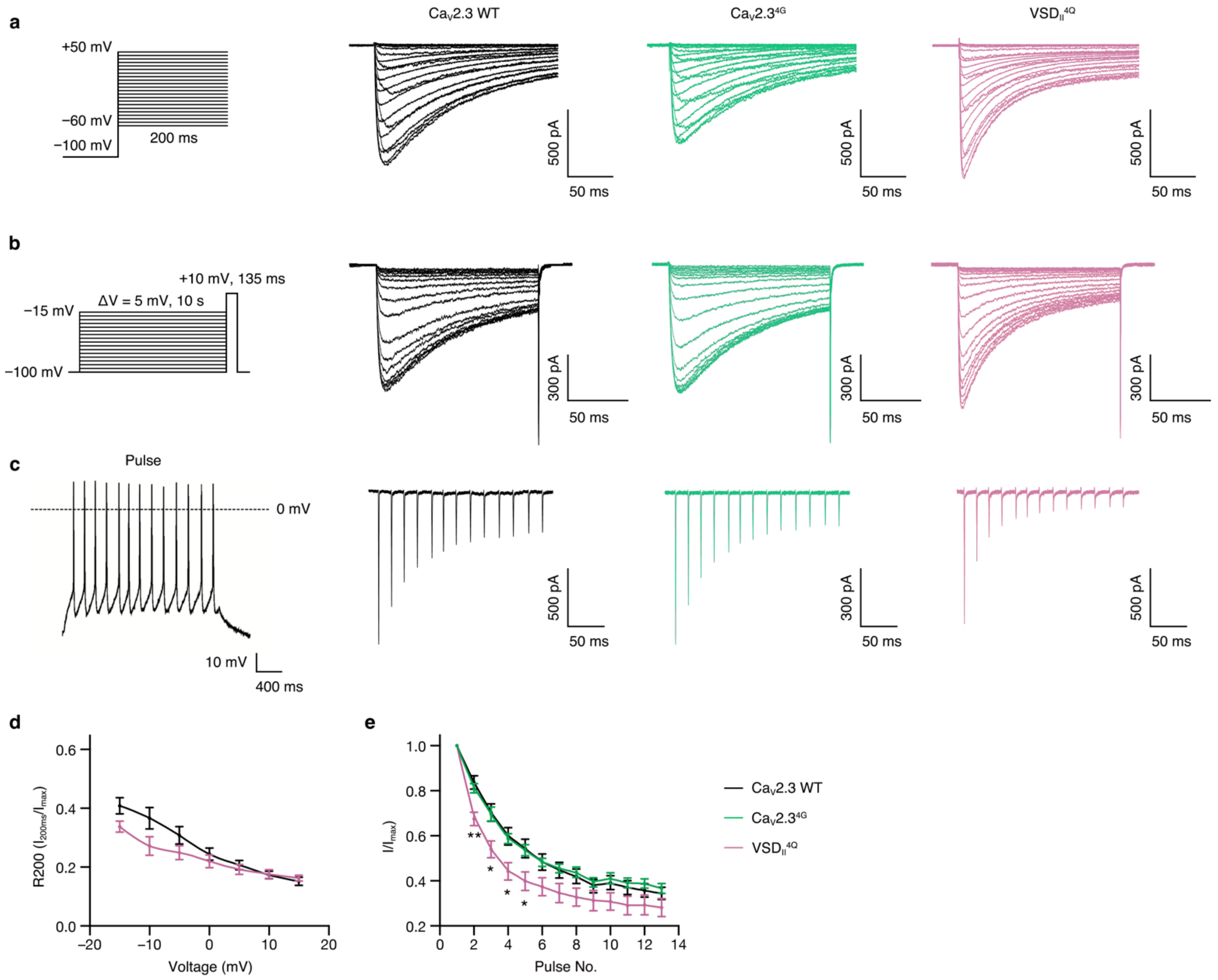

Supplementary Figure 5. Representative whole-cell current traces for VSD-related mutants

Voltage-clamp protocols (left) and representative current traces for electrophysiological studies on the voltage-sensing domains (VSDs). **a**. Protocol and representative whole-cell voltage-clamp $Ca_V2.3$ currents (activation curves). Current traces were obtained from a series of 200-ms voltage steps from –60 mV to +50 mV, in 5-mV increments. Ratio of open-state inactivation (R200) were measured using 200-ms test pulses at +10 mV. **b**. Protocols and representative currents for inactivation curves. Currents were elicited by a +10-mV test pulse after holding-voltages from –100 mV to –15 mV, in 5-mV increments. **c**. Representative current responses stimulated by action potential (AP) trains (left). The AP trains were recorded using a whole-cell current-clamp from a mouse hippocampal CA1 pyramidal neuron after current injection (see Method section for the literature reference). **d**. R200 analysis of the mutants. $Ca_V2.3$ WT (black), n = 10; VSD_{II}^{4Q} (pink), n = 7. **e**. Inactivation ratio of the mutants quantified using the current density (I) elicited by each spike of AP trains divided by the maximum current (I_{max}) elicited by the first spike. $Ca_V2.3$ WT (black), n = 13; $Ca_V2.3^{4G}$ (green), n = 7; VSD_{II}^{4Q} (pink), n = 8. Significances were determined using two-sided, unpaired *t*-test. P values, $Ca_V2.3$ WT vs. VSD_{II}^{4Q}; 0.002 (No. 2), 0.01 (No. 3), 0.02 (No. 4), and 0.03 (No. 5). Data are presented as mean ± SEM. n, biological independent cells.

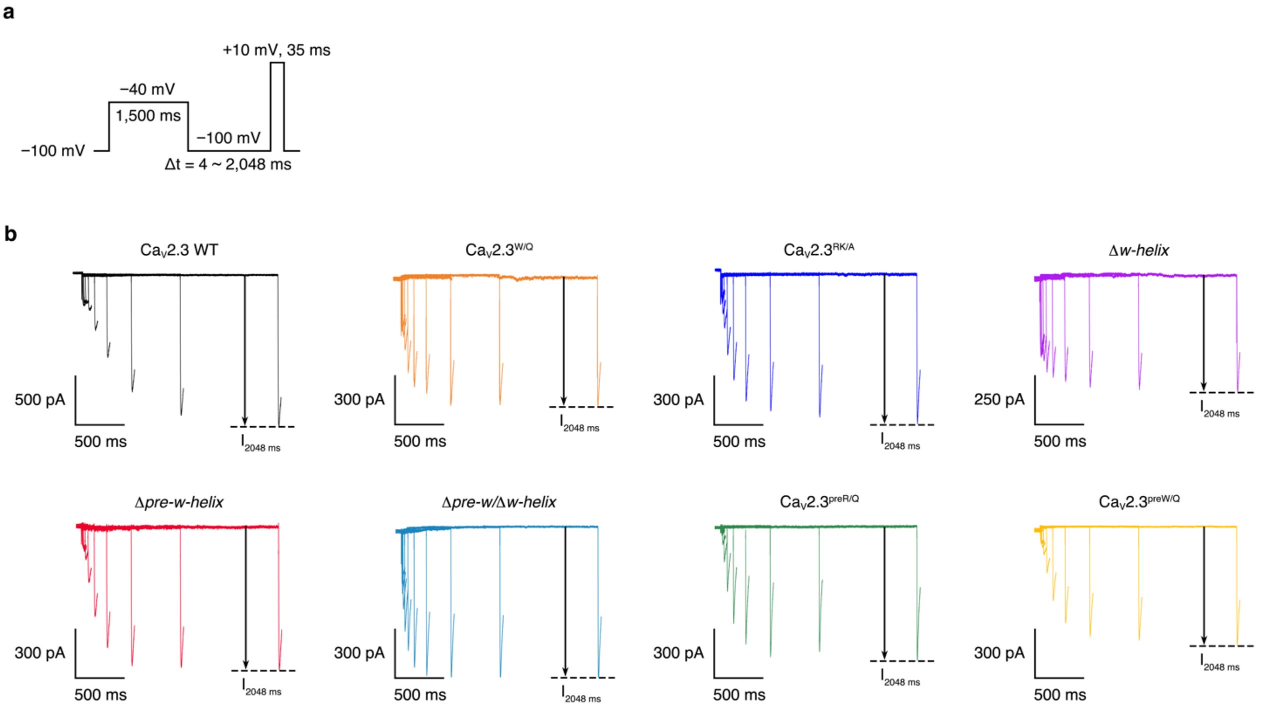

Supplementary Figure 6. Representative whole-cell current traces for CSI-related mutants

Voltage-clamp protocol (**a**) and representative current traces (**b**) of the electrophysiological studies on the recovery rate from closed-state inactivation (CSI). HEK 293-T cells expressing $Ca_V2.3$ complex were depolarized using a –40 mV pre-pulse for 1,500 ms to inactivate the channels. A recovery hyperpolarization steps to –100 mV were subsequently applied for the indicated period (4–2,048 ms), followed by a 35-ms test pulse at +10 mV.

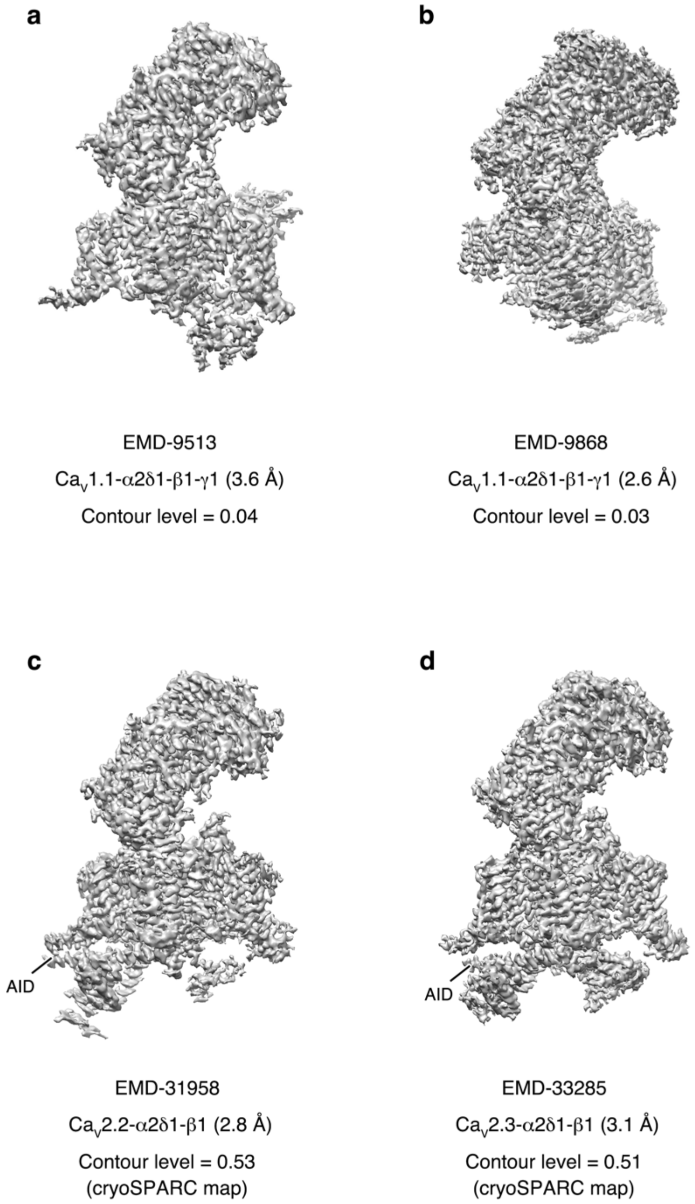

Supplementary Figure 7. Conformation heterogeneity of the AID

The alpha-interacting domains (AIDs) adopts a highly-dynamic conformation in the $Ca_V1.1$ structures (**a**–**b**) while were stabilized near the membrane plane in the intracellular side of the $Ca_V2.2$ structure (**c**) and the $Ca_V2.3$ structure (**d**) resolved in this study. The EM maps were shown as grey surfaces. Densities representing AIDs were labeled.

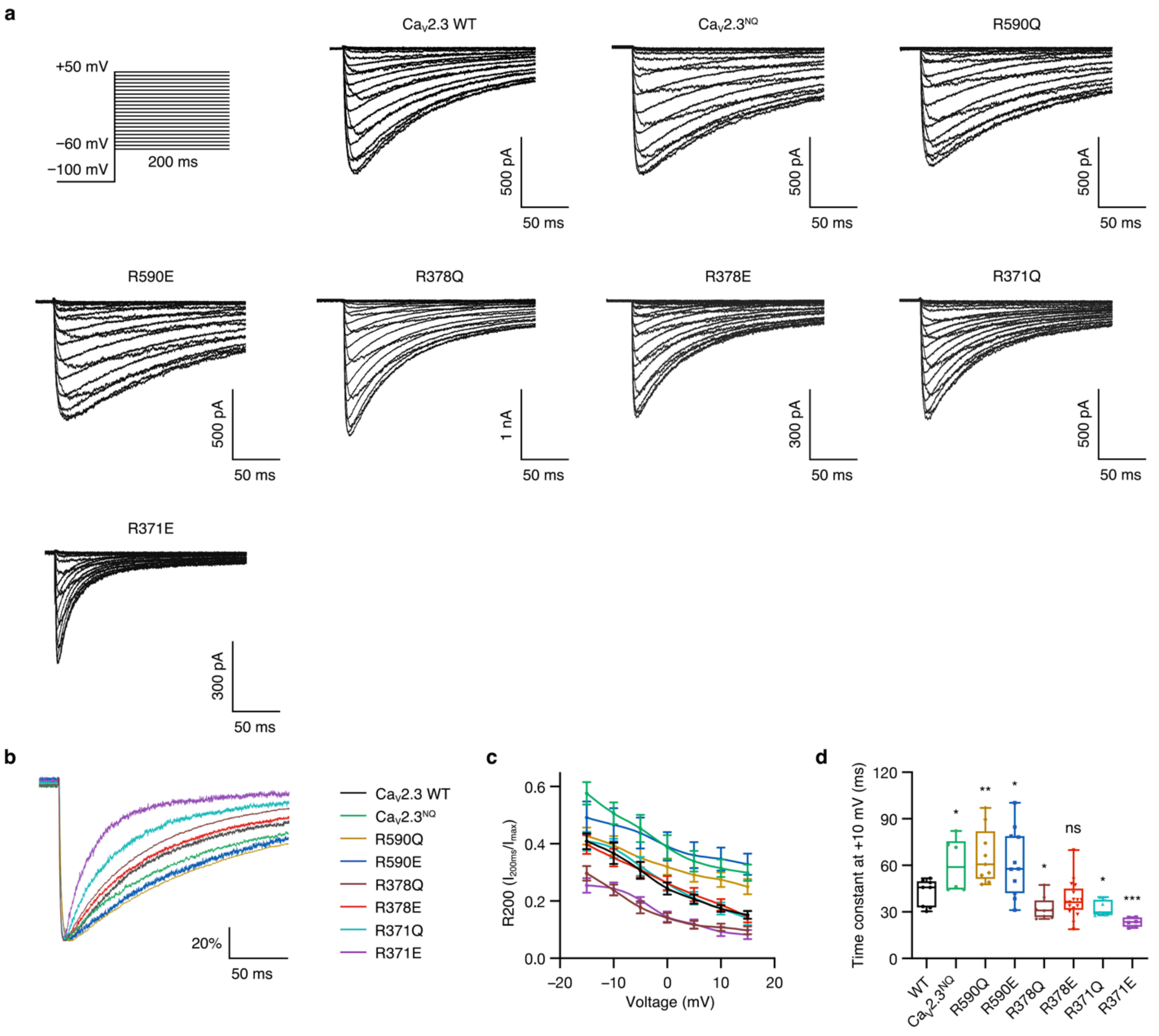

Supplementary Figure 8. Representative whole-cell current traces for OSI-related mutants

a. Voltage-clamp protocols and representative whole-cell current traces for electrophysiological studies on open-state inactivation (OSI). Current traces were obtained from a series of 200-ms voltage steps from –60 mV to +50 mV, in 5-mV increments. **b**. Representative whole-cell current traces of the OSI-related mutants under 200-ms test pulses at +10 mV. **c**. Ratio of OSI (R200) under the test pulse series ranging from –15 mV to 15 mV, measured using the current at the end of the 200-ms test pulse divided by the peak amplitude. $Ca_V2.3$ WT, n = 10; $Ca_V2.3^{NQ}$, n = 10; R590Q, n = 10; R590E, n = 6; R378Q, n = 9; R378E, n = 10; R371Q, n = 9; R371E, n = 7. Data are presented as mean ± SEM. **d**. Single-term exponential fitting of the current traces under 200-ms test pulses at +10 mV. Data are plotted as box plots. The box encompasses the interquartile range (25th–75th percentile). Whiskers illustrates the minima and maxima of the values. Mean values and medians are indicated using plus signs and dashes, respectively. $Ca_V2.3$ WT, n = 8; $Ca_V2.3^{NQ}$, n = 6; R590Q, n = 9; R590E, n = 11; R378Q, n = 8; R378E, n = 17; R371Q, n = 7; R371E, n = 6. Significances were determined using two-sided, unpaired *t*-test. P values, $Ca_V2.3$ WT vs. mutants; 0.02 ($Ca_V2.3^{NQ}$), 0.003 (R590Q), 0.03 (R590E), 0.03 (R378Q), 0.02 (R371Q), and 0.0003 (R371E). n, biological independent cells.

Supplementary Table 1.

Cryo-EM data collection, refinement, and validation statistics

	Cav2.3-α2δ1-β1 (EMD-33285) (PDB 7XLQ)
**Data collection and processing**	
Magnification	×130,000
Voltage (kV)	300
Electron exposure (e^{-}/Å^2)	60
Defocus range (μm)	−1.2 – −2.2
Pixel size (Å)	1.04
Symmetry imposed	C1
Initial particle images (no.)	787,518
Final particle images (no.)	257,473
Map resolution (Å)	3.1
FSC threshold	0.143
Map resolution range (Å)	2.5 – 4.5
**Refinement**	
Initial model used (PDB code)	7VFS
Model resolution (Å)	3.3
FSC threshold	0.5
Map sharpening *B* factor (Å^2)	−94.7
Model composition	
Non-hydrogen atoms	20,247
Protein residues	2,436
Ligands	36
B factors (Å^2)	
Protein	67.32
Ligand	53.85
R.m.s. deviations	
Bond lengths (Å)	0.006
Bond angles (°)	0.805
Validation	
MolProbity score	2.03
Clashscore	10.84
Poor rotamers (%)	0.23
Ramachandran plot	
Favored (%)	92.38
Allowed (%)	7.58
Disallowed (%)	0.04

Supplementary Table 2. Primers used in this study

Name	Sequence
$Ca_V2.3$-F	atggctcgcttcggggaggcgg
$Ca_V2.3$-R	gcatttgtcatcttcttccgtgtcacttag
$Ca_V2.3^{4G}$-F	tgtgagggtggcggtggcgcaccatcagggcagaacgagaa
$Ca_V2.3^{4G}$-R	gatggtgcgccaccgccaccctcacagcccttctccccaa
VSD_{II}^{4Q}-F	tccAgcttctaCAaatatttCAaataaccaagtattgggcttcc
VSD_{II}^{4Q}-R	aatattTGtagaagcTggagggctTgcaagacactgattcc
delta-w-helix-F	gagcggaggcgcatgcagatgtccagccaggaggccctca
delta-w-helix-R	cctggctggacatctgcatgcgcctccgctccctcaggtggctg
delta-pre-w-helix-F	aagagacagaaggcgccggcaccacatgtccgtgtgggagca
delta-pre-w-helix-R	gtgccggcgccttctgtctctttcgatcgaaggcatgttggg
delta-pre-w/w-helix-F	agagacagaaggagaatgcagatgtccagccaggaggccctc
delta-pre-w/w-helix-R	gacatctgcattctccttctgtctctttcgatcgaaggcatg
$Ca_V2.3^{W/Q}$ (W778Q)-F	acatgtccgtgCAggagcagcgtaccagccagctgaggaagca
$Ca_V2.3^{W/Q}$ (W778Q)-R	gctgctccTGcacggacatgtggtgccggcgc
$Ca_V2.3^{PreW/Q}$ (W759Q)-F	tcgatgCAggagccacgcagcagcc
$Ca_V2.3^{PreW/Q}$ (W759Q)-R	gtggctccTGcatcgacatgtggtgtct
$Ca_V2.3^{RK/A}$-F	agcagGCtaccagccagctgGCgGCgcacatgcagatgtccag
$Ca_V2.3^{RK/A}$-R	tgcGCcGCcagctggctggtaGCctgctcccacacggacatgtgg
$Ca_V2.3^{PreR/Q}$-F	tgtgggagccacAGagcagccacctgCAggagCAgaggcgccg
$Ca_V2.3^{PreR/Q}$-R	aggtggctgctCTgtggctcccacatcgacatgtggtgtTGtctccttc
$Ca_V2.3^{NQ}$-F	aactgaccaagAatAATcagAATAACAATAACgcc
$Ca_V2.3^{NQ}$-R	TctgATTatTcttggtcagttcctgggcgttgg
R590E-F	tccctaGAgaatttggtggtctccttgat
R590E-R	ccaaattcTCtagggaagcccaatacttg
R590Q-F	tccctaCAgaatttggtggtctccttgat
R590Q-R	ccaaattcTGtagggaagcccaatacttg
R371E-F	tcatgaagctgGAgcgccagcagcagat
R371E-R	gcgcTCcagcttcatgaaagcccttcg
R371Q-F	tcatgaagctgCAgcgccagcagcagat
R371Q-R	gcgcTGcagcttcatgaaagcccttcg
R378E-F	gattgagGAAgagctgaatggctaccgtgc
R378E-R	tcagctcTTCctcaatctgctgctgg
R378Q-F	gattgagCAAgagctgaatggctaccgtgc
R378Q-R	tcagctcTTGctcaatctgctgctgg
