# Supplementary material for: Molecular insights into the gating mechanisms of voltage-gated calcium channel CaV2.3
Source: Nat Commun. 2023 Jan 31;14:516. doi: 10.1038/s41467-023-36260-2 (PMC9889812; doi:10.1038/s41467-023-36260-2)
Supplement: Supplementary file 3 — Reporting Summary [file 41467_2023_36260_MOESM3_ESM.pdf]

nature portfolio

Corresponding author(s): Zhuo Huang, Yan Zhao

Last updated by author(s): Jan 14, 2023

Reporting Summary

Nature Portfolio wishes to improve the reproducibility of the work that we publish. This form provides structure for consistency and transparency in reporting. For further information on Nature Portfolio policies, see our Editorial Policies and the Editorial Policy Checklist.

Statistics

For all statistical analyses, confirm that the following items are present in the figure legend, table legend, main text, or Methods section.

n/a	Confirmed	
☐	☒	The exact sample size (*n*) for each experimental group/condition, given as a discrete number and unit of measurement
☐	☒	A statement on whether measurements were taken from distinct samples or whether the same sample was measured repeatedly
☐	☒	The statistical test(s) used AND whether they are one- or two-sided<br>*Only common tests should be described solely by name; describe more complex techniques in the Methods section.*
☒	☐	A description of all covariates tested
☒	☐	A description of any assumptions or corrections, such as tests of normality and adjustment for multiple comparisons
☐	☒	A full description of the statistical parameters including central tendency (e.g. means) or other basic estimates (e.g. regression coefficient) AND variation (e.g. standard deviation) or associated estimates of uncertainty (e.g. confidence intervals)
☐	☒	For null hypothesis testing, the test statistic (e.g. *F*, *t*, *r*) with confidence intervals, effect sizes, degrees of freedom and *P* value noted<br>*Give P values as exact values whenever suitable.*
☒	☐	For Bayesian analysis, information on the choice of priors and Markov chain Monte Carlo settings
☒	☐	For hierarchical and complex designs, identification of the appropriate level for tests and full reporting of outcomes
☒	☐	Estimates of effect sizes (e.g. Cohen's *d*, Pearson's *r*), indicating how they were calculated

Our web collection on statistics for biologists contains articles on many of the points above.

Software and code

Policy information about availability of computer code

Data collection	SerialEM v3.8, PATCHMASTER v2x90, Clampfit v10.7
Data analysis	MotionCor2 v1.4.7, Gctf v1.18, cryoSPARC v3.3.2, RELION v3.1.3, PHENIX v1.18.2, Coot v0.9.8.1, PyMOL v2.5.2, UCSF Chimera v1.16, UCSF ChimeraX v1.3, HOLE v2.2.005, Origin v2019b, Prism v9.1.1

For manuscripts utilizing custom algorithms or software that are central to the research but not yet described in published literature, software must be made available to editors and reviewers. We strongly encourage code deposition in a community repository (e.g. GitHub). See the Nature Portfolio guidelines for submitting code & software for further information.

Data

Policy information about availability of data

All manuscripts must include a data availability statement. This statement should provide the following information, where applicable:

- Accession codes, unique identifiers, or web links for publicly available datasets
- A description of any restrictions on data availability
- For clinical datasets or third party data, please ensure that the statement adheres to our policy

The cryo-EM density map of the CaV2.3-alpha2/delta1-beta1 complex has been deposited in the Electron Microscopy Data Bank (EMDB) under the accession code EMD-33285 [https://www.ebi.ac.uk/pdbe/entry/emdb/EMD-33285].

The coordinate for the CaV2.3 complex has been deposited in the Protein Data Bank (PDB) under the PDB ID 7XLQ [http://doi.org/10.2210/pdb7XLQ/pdb].

The starting model used to build CaV2.3-alpha2/delta1-beta1 is available in the PDB under the PDB ID 7VFS [http://doi.org/10.2210/pdb7VFS/pdb] (CaV2.2-alpha2/delta1-beta1).

DNA sequences of human CaV2.3 alpha1E, human alpha2/delta1, and human beta1 are available in the Universal Protein Resource (UniProt) databases under accession codes and links below:
Q15878-1 (CACNA1E isoform 1) [https://www.uniprot.org/uniprotkb/Q15878/entry]
P54289 (CACNA2D1) [https://www.uniprot.org/uniprotkb/P54289/entry]
Q02641 (CACB1) [https://www.uniprot.org/uniprotkb/Q02641/entry]

Source data including uncropped scan of the gel image and values of all electrophysiological experiment graphs are provided as Source Data Files.

Human research participants

Policy information about studies involving human research participants and Sex and Gender in Research.

Reporting on sex and gender	N/A
Population characteristics	N/A
Recruitment	N/A
Ethics oversight	N/A

Note that full information on the approval of the study protocol must also be provided in the manuscript.

Field-specific reporting

Please select the one below that is the best fit for your research. If you are not sure, read the appropriate sections before making your selection.

☒ Life sciences ☐ Behavioural & social sciences ☐ Ecological, evolutionary & environmental sciences

For a reference copy of the document with all sections, see nature.com/documents/nr-reporting-summary-flat.pdf

Life sciences study design

All studies must disclose on these points even when the disclosure is negative.

Sample size	For the cryo-EM analysis, the number of micrographs is determined by the available microscope time.<br><br>For the electrophysiology analysis, n > 6 biological independent cells was used for all experiments. Similar sample sizes have been reported in previous electrophysiological studies on CaV channels (10.1016/j.celrep.2021.109931 and 10.1038/s41467-022-29728-0) and are usually robust for statistical analysis.
Data exclusions	No data were excluded from analysis.
Replication	Sample preparation-related experiments including protein purification and SDS-PAGE gel electrophoresis were reproduced at least three times independently. Whole-cell patch clamp recording were reproduced with about 6–10 different cells. All attempts at replication were successful.
Randomization	Randomization is not relevant to cryo-EM, electrophysiology, and any other experiments in this study, because the samples were not allocated into experimental groups during data acquisition and analysis.
Blinding	Blinding is not relevant to this study. The parameters for biochemistry, cryo-EM, electrophysiology, and any other experiments in this study did not require subjective assessments of the treatments.

Reporting for specific materials, systems and methods

We require information from authors about some types of materials, experimental systems and methods used in many studies. Here, indicate whether each material, system or method listed is relevant to your study. If you are not sure if a list item applies to your research, read the appropriate section before selecting a response.

Materials & experimental systems

n/a	Involved in the study
☒	☐ Antibodies
☐	☒ Eukaryotic cell lines
☒	☐ Palaeontology and archaeology
☒	☐ Animals and other organisms
☒	☐ Clinical data
☒	☐ Dual use research of concern

Methods

n/a	Involved in the study
☒	☐ ChIP-seq
☒	☐ Flow cytometry
☒	☐ MRI-based neuroimaging

Eukaryotic cell lines

Policy information about cell lines and Sex and Gender in Research

Cell line source(s)	FreeStyle 293-F cells (Gibco, USA); 293-T cells (Gibco, USA); Sf9 cells (Gibco, USA)
Authentication	The cell lines were not authenticated.
Mycoplasma contamination	The cells were tested negative for mycoplasma contamination.
Commonly misidentified lines (See ICLAC register)	No commonly misidentified lines were used.
