# Supplementary figures and images for: Molecular insights into the gating mechanisms of voltage-gated calcium channel CaV2.3
Source: Nat Commun. 2023 Jan 31;14:516. doi: 10.1038/s41467-023-36260-2 (PMC9889812; doi:10.1038/s41467-023-36260-2)

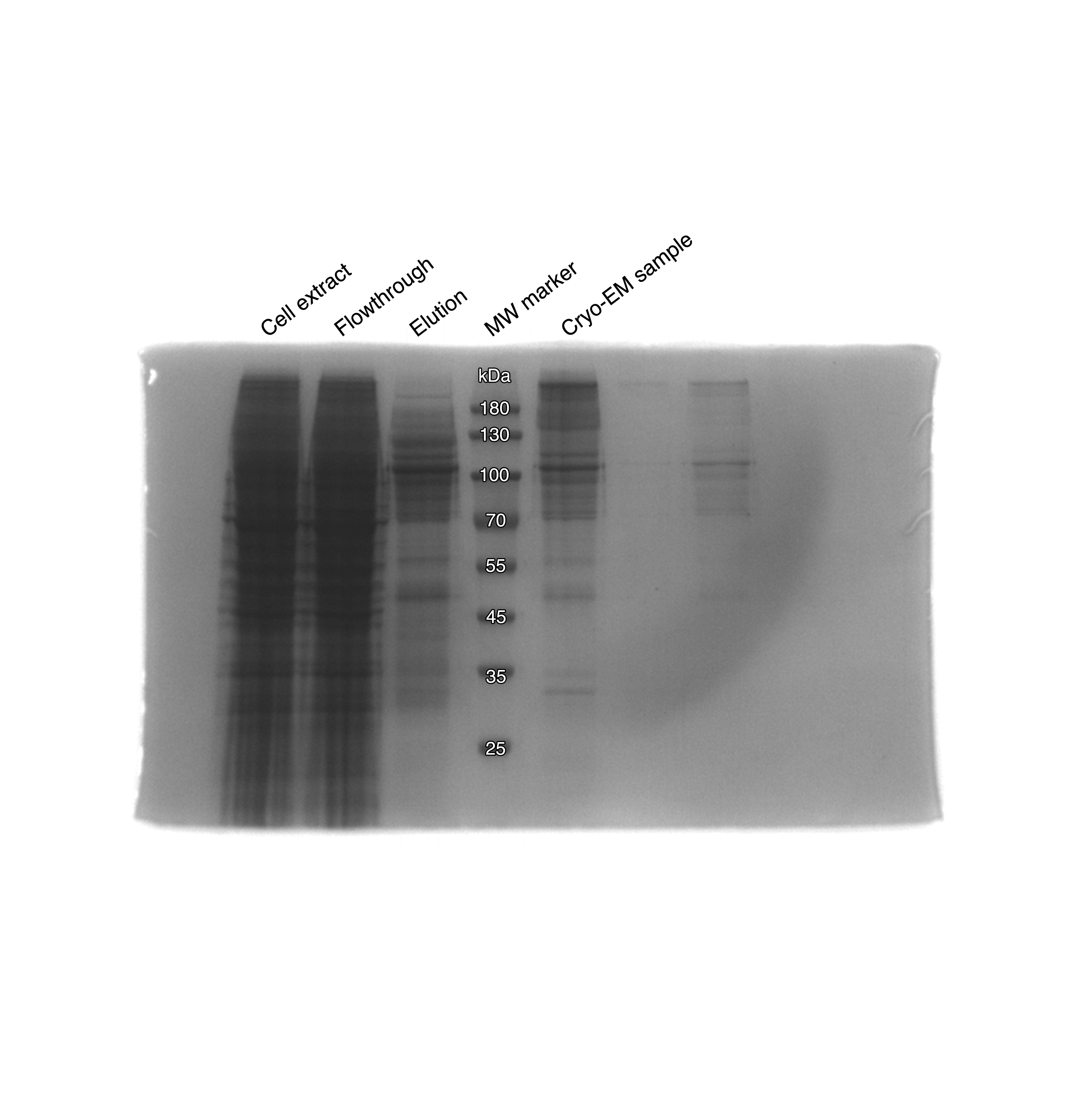

Supplement: Supplementary file 4 — Source Data [file 41467_2023_36260_MOESM4_ESM.zip › Source Data Files/Source_Data_1-Supplementary_Figure_1b.jpg]
